# Hepatobiliary disease caused by *Henneguya* sp. (Cnidaria:Myxozoa) in an Amazonian fish *Chaetobranchopsis orbicularis* (Characidae)

**DOI:** 10.1590/S1984-29612025023

**Published:** 2025-05-23

**Authors:** José Ledamir Sindeaux-Neto, Amanda Queiroz Azevedo, Natalia dos Anjos, Camila Maria Barbosa Pereira, Jhonata Eduard, Michele Velasco

**Affiliations:** 1 Laboratório de Integração Morfo-Molecular e Tecnologias – LIMT, Instituto da Saúde e Produção Animal, Universidade Federal Rural da Amazônia – UFRA, Belém, PA, Brasil; 2 Programa de Pós-graduação em Reprodução Animal na Amazônia – REPROAMAZON, Universidade Federal Rural da Amazônia – UFRA, Belém, PA, Brasil; 3 Programa de Pós-graduação Rede de Biodiversidade e Biotecnologia da Amazônia Legal – BIONORTE, Universidade Federal do Pará – UFPA, Belém, PA, Brasil; 4 Programa de Pós-graduação em Biologia de Agentes Infecciosos e Parasitários – PPGBAIP, Universidade Federal do Pará – UFPA, Belém, PA, Brasil; 5 Programa de Pós-graduação em Saúde e Produção Animal na Amazônia – PPGSPAA, Universidade Federal Rural da Amazônia – UFRA, Belém, PA, Brasil

**Keywords:** Henneguyosis, liver, gallbladder, histopathology, Marajó island, Henneguyose, fígado, vesícula biliar, histopatologia, ilha do Marajó

## Abstract

The present study describes multifocal infection by *Henneguya* sp. in the liver and gallbladder of *Chaetobranchopsis orbicularis* Steindachner 1875, from the Arari River, on Marajó Island, in the Brazilian Amazon. Fifty specimens of *C. orbicularis* were examined, and after parasitic confirmation, small fragments of the infected organs were removed, fixed in Davidson’s solution, and histologically processed. Multifocal infection in the liver and gallbladder was observed in 56% (28/50) of *C. orbicularis* specimens. The cysts were full of mature spores with morphological characteristics inherent to the genus *Henneguya*, with the same morphological and morphometric characteristics: body elongated, ellipsoidal shape in frontal view, and a thick, translucent, birefringent hyaline sheath covering the entire structure of the spore. Histopathological analysis showed cysts with a predominantly fibroblastic capsule, containing spores inside, with a loss of cellular architecture in the liver parenchyma, as well as deformation of the gallbladder mucosal epithelium. Although clinical signs were not observed, the histopathological findings presented alterations that characterize henneguyosis, a microparasitic disease the host, from the Amazon region.

## Resumo

O presente estudo descreve a infecção multifocal, causada por *Henneguya* sp. no fígado e na vesícula biliar de *Chaetobranchopsis orbicularis* Steindachner 1875, rio Arari, ilha do Marajó, Amazônia brasileira. Cinquenta espécimes de *C. orbicularis* foram examinados, e após a confirmação do parasitismo, retiraram-se pequenos fragmentos dos órgãos infectados, fixados em solução de Davidson, e processados para a histologia. Em 56% (28/50) dos espécimes de *C. orbicularis* foram observados infecção multifocal no fígado e na vesícula biliar. Os cistos estavam repletos de esporos maduros com características morfológicas inerentes ao gênero *Henneguya*, com as mesmas características morfológicas e morfométricas: corpo alongado, formato elipsoidal em vista frontal, e bainha hialina espessa, translúcida, birrefringente, cobrindo toda a estrutura do esporo. A análise histopatológica revelou cistos com cápsula predominantemente fibroblástica, contendo esporos em seu interior, com perda da arquitetura celular no parênquima hepático, além de deformação do epitélio da mucosa da vesícula biliar. Embora não tenham sido observados sinais clínicos, os achados necroscópicos apresentaram alterações que caracterizam a henneguyose, doença microparasitária em hospedeiro da região amazônica.

## Introduction

The rivers of the Amazon basin are home to the largest ichthyofauna in the world, with more than 2.257 described species, 1.248 of which are endemic and represent approximately 15% of the world’s freshwater fish identified to date (Oberdorff et al., 2019). These animals have a variety of pathogens, but owing to their nutritional and physiological states being in balance with the environment, they do not show clinical signs except when there are alterations in this balance, mainly of nature environmental, which can lead to the manifestation of some diseases (Pavanelli et al., 2002; Buchmann, 2022).

Parasitic diseases are prevalent in fish because of the peculiarities of the aquatic environments that facilitate their propagation, reproduction, and life cycles (Feist & Longshaw, 2006; Fiala et al., 2015). Among the most important parasites in aquatic animals, we highlighted the Myxozoa Class with 62 genera, represented by spore-producing species parasitizing various vertebrates and causing diseases in freshwater and marine fish, which can cause high mortality rates in species of different geographical areas (Griffin et al., 2008).

Members of the genus *Henneguya,* are important fish pathogens that cause henneguyosis. Belonging to the order Bivalvulida, it is the second largest genus in the Myxobolidae family, parasitizing mainly freshwater Neotropical fish, with descriptions of new species occurring regularly (Rangel et al., 2023; Adriano & Eiras, 2024).

The Acará Branco, *Chaetobranchopsis orbicularis* Steindachner 1875, is an omnivorous cichlid with a wide distribution in South America and can be found in the Amazon basin, from the Rio Negro to the Marajó Island, and in Amapá (Kullander, 2003). This species is commercially important for extractive fishing and exhibits environmental tolerance characteristics that make it suitable for aquaculture cultivation (Ardura et al., 2010; Tavares-Dias & Oliveira, 2017; Souza et al., 2021).

This study describes a multifocal infection by *Henneguya* sp. in the liver and gallbladder of the cichlid *C. orbicularis*, from the Arari River on Marajó Island, in the Brazilian Amazon.

## Materials and Methods

Fifty specimens adult and juvenile specimens of *C. orbicularis* (25 females and 25 males), were examined, measuring 15-20 cm and weighing ~25-30 g, captured with cast net and gillnet in the Arari River municipality of Cachoeira do Arari (1°00’ S, 48°57” W), located in Marajó Island state of Pará, Brazil. The specimens were transported alive in plastic bags with water from the natural environment and artificial aeration to the municipality of Salvaterra, and then to the Morpho-molecular Integration and Technologies Laboratory (LIMT) at the Federal Rural University of Amazonia (UFRA/Belém) where they were kept alive in aquariums. The animals were anesthetized with tricaine methanesulfonate (MS222, Sigma) at a concentration of 50 mg/L (CEUA 13/2014) and dissected for parasite and cyst detection using a stereomicroscope for observation and analysis.

The presence of cysts and spores in the tegument, gills, coelomic cavity and internal organs was analyzed. Small fragments (up to 0.5 centimeters thick) were removed for observation under light microscopy (LM), fixed in Davison’s solution (neutral buffered formalin, glacial acetic acid, ethyl alcohol 95%, distilled water) for 24 h; subsequently, the fragments were histologically processed, embedded in paraffin, sectioned into 5 µm thick slices and stained using the Hematoxylin-Eosin and Ziehl–Neelsen techniques (Luna, 1968).

Photomicrographs were captured using a Zeiss AxioCam ERc 5s camera, attached to a Zeiss Primo Star microscope, to measure the spores (n=30) using AxioVision LE software. A microscope with differential interference contrast (Nomarski-DIC) was used to visualize the spore structures.

## Results and Discussion

Small whitish cysts were observed in the liver and gallbladder in 28 adults specimens (56%), 14 males and 14 females. The cysts were filled with mature spores with morphological characteristics inherent to the *Henneguya* genus. These findings contrast previous research, which demonstrated that *Henneguya* infections were higher in juvenile fish due to their immature immune systems, making them more susceptible to parasites (Smith & Inslee, 1980). Regarding the sites of infection, it is possible that it occurred as a result of dietary ingestion of spores or cysts, since *C. orbicularis* is an omnivorous species and has been shown to have a high diversity of endoparasites when compared to detritivorous and piscivorous fish (Baia et al., 2018). Another aspect that may influence prevalence is seasonality, where cysts may rupture in warmer periods and the number of infected species may increase (Cone, 1994). However, it would be necessary to increase the sampling period, since the samples collected in this study only occurred in July, the beginning of summer on the Amazon.

It was observed that larger specimens presented higher parasite loads, this was also observed for ectoparasites in *C. orbicularis* (Tavares-Dias & Oliveira, 2017). Although more prevalent, a greater intensity of infection was not observed, a similar result was described in the infection of *Zschokkella leptatherinae* that in the hepatic ducts and gallbladder of *Atherinosoma microstoma* and *Leptatherina presbyteroides* (Su & White, 1996).

**Figure 1:**
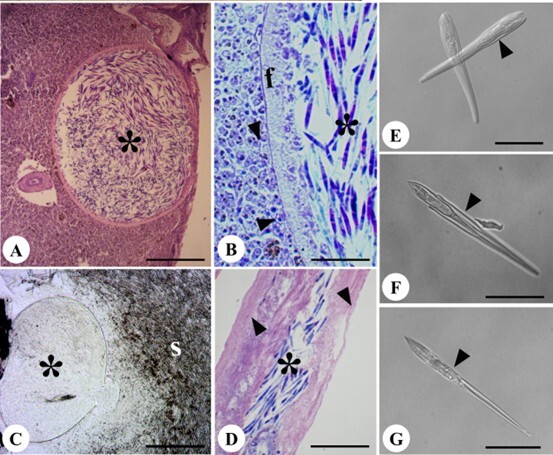
Light micrograph of *Henneguya* spores and cysts in *Chaetobranchopsis orbicularis* from the Marajó Island, in Brazilian Amazon. (A) Histological section of the liver with *Henneguya* sp. cyst. 2 (*), revealing the end of the cyst wall in contact with the Glisson’s capsule. Hematoxylin-eosin, Bar. Esc. 180 µm; (B) Histological section of the liver with *Henneguya* sp, cyst 2 (*), showing the fibrous wall of the cyst (f) and hepatocyte adjacent to the cyst (arrowhead), Ziehl–Neelsen. Bar. Esc. 40 µm; (C) Cyst of *Henneguya* sp. 2 fresh (*) located in the gallbladder, with spores dispersed in bile content (S), Bar. Esc. 210 µm; (D) Histological slice of gallbladder with cyst (*) causing epithelial deformation (arrowhead). Bar = 55 µm; (E), (F) and (G): Differential Interference Contrast of *Henneguya* sp. spores. 2, (E) within the hyaline sheath (arrowhead); (F)- emerging from the hyaline sheath (arrowhead), highlighting the polar capsules (pc); and (G) outside the hyaline sheath (arrowhead), highlighting the caudal extension (arrow). Bar Esc. are 20 µm, 20 µm, and 16 µm, respectively.

Light microscopy analyses revealed well-defined cysts containing numerous spores and spores dispersed in bile fluid (Figures 1A to 1D). The spores exhibited the same morphological and morphometric characteristics, being elongated and ellipsoidal in the frontal view, with two elongated valves, a long, tapering caudal extension, and a thick, translucent, birefringent hyaline sheath covering the entire spore structure (Figures 1E, 1F and 1G), similar to the characteristics described for the species *Henneguya torpedo* Azevedo, Casal, Matos, Alves & Matos 2011.

Several species of the genus *Henneguya*, described from the state of Pará in the Brazilian Amazon, were considered for comparison of their measurements. The species in the present study had a mean and standard deviation with a total spore length of 49.7 ± 3.2 μm, spore body length of 17.4 ± 1.1 μm, spore width of 6.0 ± 0.2 μm, caudal appendage length of 32.2 ± 2.6 μm, and polar capsule length of 8.2 ± 0.2 μm and width of 1.9 ± 0.1 μm (Table 1).

**Table 1:** Comparison of measurements of spores of *Henneguya* sp. of *Chaetobranchopsis orbicularis* from the Marajó Island, in Brazilian Amazon.

**Parasite**	**Site of Infection**	**LS**	**LSB**	**WSB**	**CA**	**PC**	**Locality**
***Henneguya* sp. (Present study)**	**Liver and gallbladder of *Chaetobranchopsis orbicularis***	**49.7 ± 3.2**	**17.4 ± 1.1**	**6.0 ± 0.2**	**32.2 ± 2.6**	**8.2 (± 0.2) x 1.9 (± 0.1)**	**River Arari/ PA Brazil**
*Henneguya malabarica* (Azevedo & Matos, 1996)	Gills of *Hoplias malabaricus*	28.3 ± 1.6	12.6 ± 0.65	8.6	17.1 ± 1.4	3.7 (± 0.65) x 1.8 (± 0.3)	River Amazonas/PA Brazil
*Henneguya astyanax* (Vita et al., 2003)	Gills of *Astyanax keithi*	47.8 ± 0.71	15.2	5.7 ± 0.71	32.6	5.0 (± 0.13) x 1.5 (± 0.07)	River Amazonas /PA Brazil
*Henneguya rhamdia* (Matos et al., 2005)	Gills of *Rhamdia quelen*	50.0 ±1.8	13.1 ± 1.1	5.2 ± 0.5	36.9 ± 1.6	4.7(± 0.4) x 1.1 (± 0.2)	River Peixe-boi /PA Brazil
*Henneguya torpedo* (Azevedo et al., 2011)	Brain and Spinal cordo of *Brachyhypopomus pinnicaudatus*	48.6 ± 0.51	28.5 ± 0.36	7.2 (7.0-7.5)	19.6 (19.2-19.9)	6.4 (± 0.26) x 1.8 (± 0.19)	River Peixe-boi /PA Brazil
*Henneguya aequidens* (Videira et al., 2015)	Gills of *Aequidens plagiozonatus*	41 ± 1.5	15 ± 0.9	6 ± 0.8	27 ± 0.6	3 ± 0.3 x 3 ± 0.3	River Peixe-boi /PA Brazil
*Henneguya paraensis* (Velasco et al., 2016)	Gills of *Cichla temensis*	42.3 ± 0.65	12.8 ± 0.42	8.6 ± 0.32	29.5 ± 0.7	7.4 (± 0.16) x 2.6 (± 0.08)	River Amazonas /PA Brazil
*Henneguya santarenensis* (Naldoni et al., 2018)	Gills of *Phractocephalus hemioliopterus*	31.9 ± 3	10.8 ± 0.5	4.3 ± 0.3	21 ± 3.1	4.6 (±0.4) x 1.4 (±0.2)	River Tapajós/PA Brazil
*Henneguya tucunarei* (Zatti et al., 2018)	Gills of *Cichla monoculus*	43.8 ± 6.75	14 ± 1.8	6.1 ± 1.45	28.1 ± 8	3.4 (±1.05) x 2(±0.65)	River Tapajós/PA Brazil
*Henneguya tapajoensis* (Zatti et al., 2018)	Gills of *Cichla pinima*	54.6 ± 7.5	16.4 ± 2.3	7 ± 1.8	39 ± 7.4	4.2 (±1.05) x 2.1 (±0.65)	River Tapajós/PA Brazil
*Henneguya* sp. (Figueredo et al., 2020)	Gills of *Metynnis hypsauchen*	61.9 ± 1.25	38.2 ± 0.8	5.8 ± 0.45	23.7 ± 0.5	6.1 (±0.15) x 1.9 (±0.1)	River Capim/PA Brazil
*Henneguya correai* (Müller et al., 2023)	Fins of *Semaprochilodus insignis*	48 ± 4.9	15 ± 1.6	4.0 ± 0.6	33.7 ± 4.5	7.2 (±0.8) x 1.5 (±0.3)	River Tapajós/PA Brazil

LS = total length of the spore; LSB = length of the spore body; WSB = width of the spore body; CA = caudal appendages; PC = polar capsules (length × width). All measurements are provided in micrometers.

The total spore length in this study showed similarity to the species *Henneguya rhamdia* Matos, Tajdari & Azevedo 2005, with 50.0 ± 1.8 µm; however, this species does not have a thick hyaline sheath on its exterior and has an ovoid to pyriform spore body. The *Henneguya correai* Müller, Figueredo, Atkinson, Bartholomew and Adriano 2023, also has total spore length similar to the study, with 48 ± 4.9 μm; however, differences are observed in specific dimensions, such as spore width and polar capsule measurements, as well as different morphological characteristics. Although there were similarities between *Henneguya* in this study and other species of the genus, nuances in dimensions and specific characteristics allowed for this distinction. Nevertheless, morphological and morphometric comparisons showed a strong similarity to the specie *H. torpedo* (Azevedo et al., 2011), with differentiation possible only by the size of their spore bodies, where *H. torpedo* had a spore body 60% larger than that of the *Henneguya* in the present study (Table 1).

Species such as *Henneguya paraensis* Velasco et al., 2016, *Henneguya santarenensis* Naldoni, Maia, Correa, Silva & Adriano 2018, and *Henneguya tucunarei* Zatti, Atkinson, Maia, Bartholomew & Adriano 2018, exhibited different morphologies and morphometries, with all having spores considerably smaller than those in this study.

Although no clinical signs of disease were observed in the hosts in this study, such as lethargy, respiratory distress, pale skin, increased mucus production, abdominal distension, swollen gills, common in *Henneguya* infections (Bowser & Conroy, 1985; Sant’Ana et al., 2012; Yassen et al., 2024; Uma et al., 2025). Histopathological analyses showed spherical cysts with fibroblastic walls in the liver, as well as deformation of the hepatic parenchyma, with loss of the architecture of adjacent hepatocyte cords. In the gallbladder, cysts were large and elongated, causing compression and deformation of the mucosal epithelium. Katharios et al. (2020) described hepatic disease in the fish *Pagrus major* Temminck and Schlegel 1843, caused by infection with *Henneguya aegea* Katharios, Varvarigos, Keklikoglou, Ruetten, Soja et al. 2020, where the host exhibited palor due to vacuolar lipidosis, petechial hemorrhages, and greenish areas possibly related to bile. Silva et al. (2018) reported that *Henneguya* causes hyperplasia and hypertrophy in the liver. In contrast, no clinical sign of disease was observed in the liver of *C. orbicularis*.

Multifocal infection in different organs has been described in previous studies. For example, *Henneguya arapaima* Feijó, Arana, Ceccarelli & Adriano 2008, parasitizing the gallbladder and the gill arch of *Arapaima gigas* Schinz 1822, while *Henneguya friderici* Casal, Matos and Azevedo 2003, was identified it in the gills, intestines, kidneys, and liver of *Leporinus friderici* Bloch, 1794.

This study describes hepatobiliary infection by *Henneguya* sp. in the liver and gallbladder, with histopathological alterations. Despite the high parasitic prevalence of henneguyosis in *C. orbicularis*, this host did not exhibit any clinical signs of this disease. This is the first report of Henneguyosis in *C. orbicularis*, an economically important fish for the Brazilian Amazon.
